# Two cases of resectable pancreatic cancer diagnosed by open surgical biopsy after endoscopic ultrasound fine-needle aspiration failed to yield diagnosis: case reports

**DOI:** 10.1186/s40792-017-0314-2

**Published:** 2017-02-25

**Authors:** Reishi Toshiyama, Takehiro Noda, Hidetoshi Eguchi, Yoshifumi Iwagami, Daisaku Yamada, Tadafumi Asaoka, Hiroshi Wada, Koichi Kawamoto, Kunihito Gotoh, Yutaka Takeda, Masahiro Tanemura, Eiichi Morii, Koji Umeshita, Masaki Mori, Yuichiro Doki

**Affiliations:** 1 Department of Gastroenterological Surgery, Graduate School of Medicine, Osaka University, 2-2 Yamadaoka E-2, Suita, Osaka 565-0871 Japan; 2 Department of Pathology, Graduate School of Medicine, Osaka University, Osaka, Japan; 3 Department of Health Sciences, Graduate School of Medicine, Osaka University, Osaka, Japan; 4 Department of Surgery, Kansai Rosai Hospital, Hyogo, Japan; 5 Department of Surgery, Osaka Police Hospital, Osaka, Japan

**Keywords:** Open surgical biopsy, Intraoperative pancreas biopsy, Pancreatic cancer

## Abstract

**Background:**

Tumor biopsy for histological diagnosis is required preoperatively and before initiating chemotherapy or radiation therapy for patients with pancreatic cancer (Cancer of the Pancreas: Clinical Practice Guidelines, European Society for Medical Oncology). Endoscopic ultrasound fine-needle aspiration (EUS-FNA) is widely applied to obtain tissue samples for histological examination. However, in some cases, EUS-FNA cannot be performed safely or tissue samples are insufficient to establish a definitive diagnosis. We present two cases of pancreatic cancer diagnosed by open surgical biopsy after EUS-FNA failed to yield a diagnosis.

**Case presentation:**

Case 1 was a 50-year-old man. Computed tomography showed a hypovascular lesion in the uncus of the pancreas. Although EUS-FNA was conducted twice, we could not collect enough quantity of tissue samples to establish a definitive diagnosis. Open surgical biopsy revealed adenocarcinoma, and the patient underwent preoperative chemoradiation therapy followed by curative operation. Case 2 was a 68-year-old man. Computed tomography showed a hypovascular tumor in the uncus of the pancreas. EUS revealed a 14-mm hypoechoic lesion, but we could not perform EUS-FNA because the superior mesenteric vein was located in the puncture line. Open surgical biopsy revealed adenocarcinoma, and the patient underwent preoperative chemoradiation therapy followed by pancreaticoduodenectomy.

**Conclusions:**

EUS-FNA is the first choice in the diagnostic modalities of pancreatic neoplasm, but open surgical biopsy is an effective diagnostic method if EUS-FNA is unsuccessful.

## Background

The prognosis for pancreatic cancer is extremely poor, and the 5-year survival rate is only approximately 5% [1]. Pancreatic cancer is the fourth most fatal cancer in Western countries [2] and the fifth most common cause of death from cancer in Japan [3]. Surgical resection remains the only potentially curative therapy [4]. However, only 20% of patients with pancreatic cancer receive curative resection because most of them show either metastatic or locally advanced disease in the asymptomatic phase [5]. In 2015, the European Society for Medical Oncology (ESMO) published clinical practice guidelines for cancer of the pancreas and delineated the process for diagnostic work-ups. They recommended that computed tomography (CT) was firstly used to determine the tumor size and precise burden, as well as arterial and venous local involvement. Tumor biopsy was indicated for patients who required a histological diagnosis of malignant disease, not only preoperatively but also before initiating chemotherapy or chemoradiation therapy (CRT). Endoscopic ultrasound (EUS) is also widely applied to obtain tissue samples from primary lesions via fine-needle aspiration (FNA). The diagnostic accuracy of EUS-FNA is approximately 90% with histological confirmation of malignancy [6–8]. The contents listed in the National Comprehensive Cancer Network Clinical Practice Guidelines in Oncology (NCCN guidelines) version 2.2016 are similar to ESMO guidelines. The NCCN guidelines also recommend that neoadjuvant chemotherapy is considered for patients with high-risk features such as highly elevated carbohydrate antigen 19-9 (CA19-9) levels, large primary tumors, large regional lymph nodes, excessive weight loss, and extreme pain. The pathological diagnosis is also recommended to be necessary before neoadjuvant chemotherapy or CRT.

However, in some patients, EUS-FNA cannot be performed safely because blood vessels or the main pancreatic duct are located along the puncture route or because the lesion cannot be detected by EUS [9, 10]. Moreover, tissue samples obtained by FNA are insufficient to establish a definitive diagnosis in some cases. The core needle biopsy (CNB) provides a sufficiently large tissue sample, and open surgical biopsy can be performed after multiple attempts such as EUS-FNA are unsuccessful [11]. Herein, we present two patients with resectable pancreatic cancer who were diagnosed by open surgical biopsy after EUS-FNA failed to establish a diagnosis.

## Case presentations

**Fig. 1:**
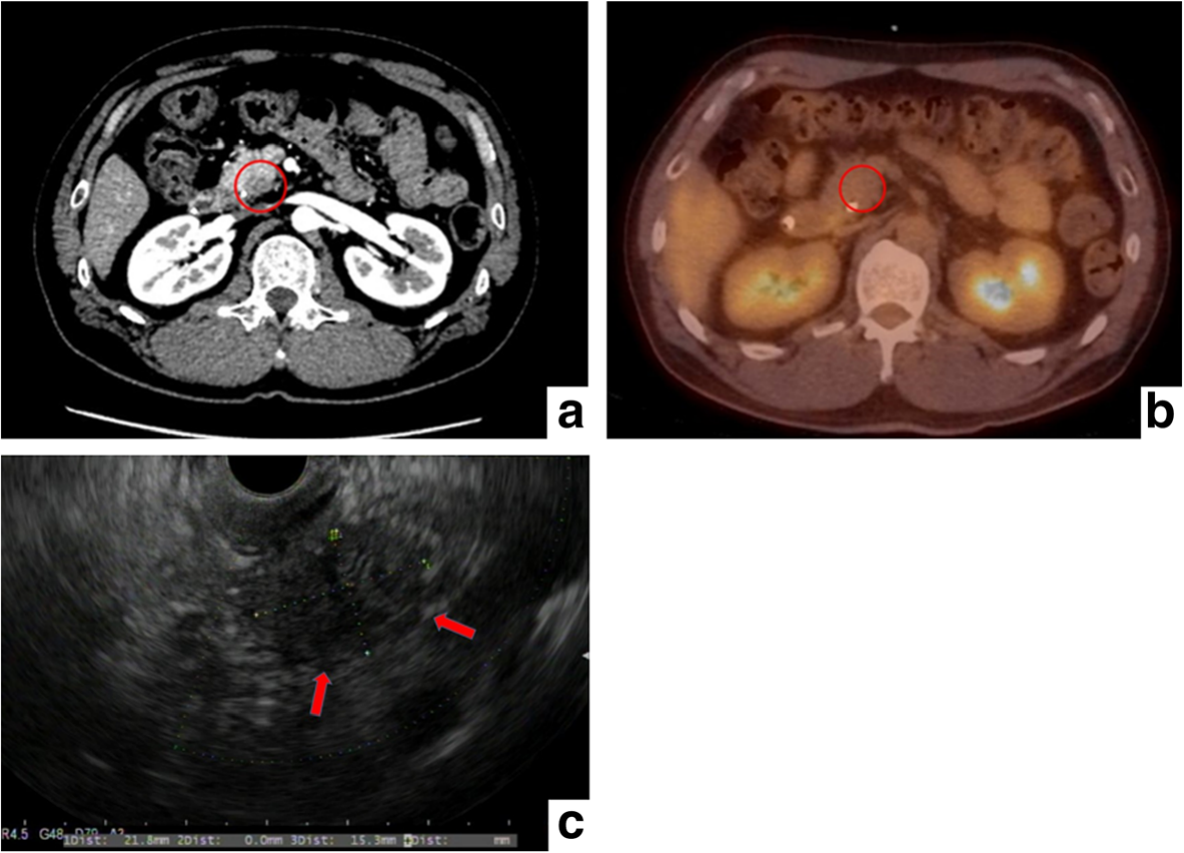
Radiological and endoscopic ultrasonographic findings for case 1. **a** CECT revealed a hypovascular tumor in the uncus of the pancreas (*red circle*). **b** PET-CT revealed that 18 F-FDG accumulation was within normal range. **c** EUS revealed a 22-mm hypoechoic lesion in the uncus of the pancreas (*red arrows*)

**Fig. 2:**
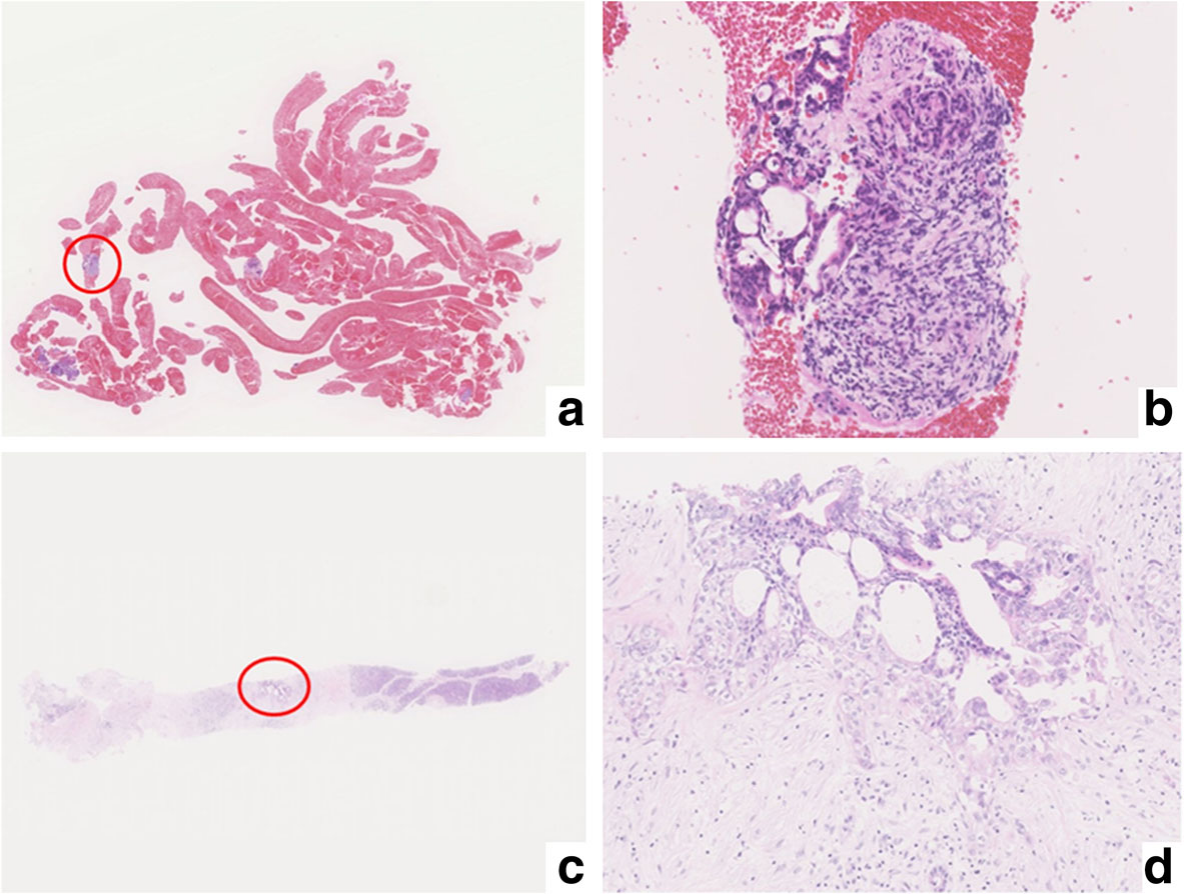
Pathological analysis by EUS-FNA compared to open surgical biopsy in case 1. **a** Macroscopic view of specimen obtained by EUS-FNA. The specimen contained large quantities of blood clots. **b** Microscopic view of specimen (×100 magnification) revealed enlarged nuclei and disordered ductal structures. **c** Specimen obtained by open surgical biopsy. Most of the specimen consisted of pancreatic parenchyma. **d** Microscopic view of specimen obtained by open surgical biopsy (×100 magnification) revealed abundant dyskaryotic cells with enlarged nuclei, disordered ductal structures

Case 1: A 50-year-old man presented to our hospital with nausea. He had elevated duke pancreatic monoclonal antigen type 2 (DUPAN-2) levels (680 U/ml), but carcinoembryonic antigen (CEA) and CA19-9 levels were within normal limits. Abdominal contrast-enhanced CT (CECT) revealed a hypovascular lesion in the uncus of the pancreas (Fig. 1a). The main pancreatic duct showed stenosis around the tumor. Positron emission tomography-CT (PET-CT) revealed that fluorine‑18 fluorodeoxyglucose (18 F-FDG) accumulation was with normal limits (Fig. 1b), but EUS detected a 22 × 15 mm hypoechoic lesion. EUS-FNA was conducted twice with a 25-gauge puncture needle (Fig. 1c). Figure 2a, b showed the specimen obtained by EUS-FNA, but the specimen contained large quantities of blood clots (Fig. 2a). Microscopically, the findings of enlarged nuclei and disordered ductal structures were observed (Fig 2b). However, the quantity of tissue sample was insufficient to diagnose adenocarcinoma. The tumor was strongly suspected to be malignant disease based on radiological findings, and so we performed intraoperative pancreas biopsy with the Bard® Magnum® Reusable Core Biopsy Instrument (C. R. Bard, Inc., Murray Hill, NJ, USA). Firstly, we used 20-gauge × 200 mm puncture needle for intraoperative biopsies, but the quantity of tumor in the samples was insufficient, so we changed this needle to thicker ones (18 gauge and 16 gauge). In performing intraoperative biopsy, the important thing was that we should puncture the mass as perpendicularly as possible. Finally, the samples obtained by needle of 16 gauge were sufficient for intraoperative diagnosis.

Intraoperative pancreas biopsy revealed that the specimen had abundant dyskaryotic cells with nuclear enlargement. Its ductal structure was disordered, and p53-positive cells were observed. These findings were consistent with a diagnosis of adenocarcinoma (Fig. 2c, d). Consequently, we performed preoperative CRT (radiation: 50.5 Gy, chemotherapy: gemcitabine and S-1) and pancreaticoduodenectomy (PD). The pathological diagnosis was invasive ductal adenocarcinoma, and the pathological stage was pT1N0M0 stage I according to the American Joint Committee on Cancer (AJCC)/Union for International Cancer Control (UICC) seventh edition staging system. The therapeutic effect of preoperative CRT was grade IIa according to the Evans classification system [12]. The patient received adjuvant chemotherapy with S-1 (120 mg/day) for 6 months after the operation. Nine months after the operation, CECT revealed regional lymph node recurrence and the patient was treated with systemic chemotherapy (gemcitabine and nab-paclitaxel).

**Fig. 3:**
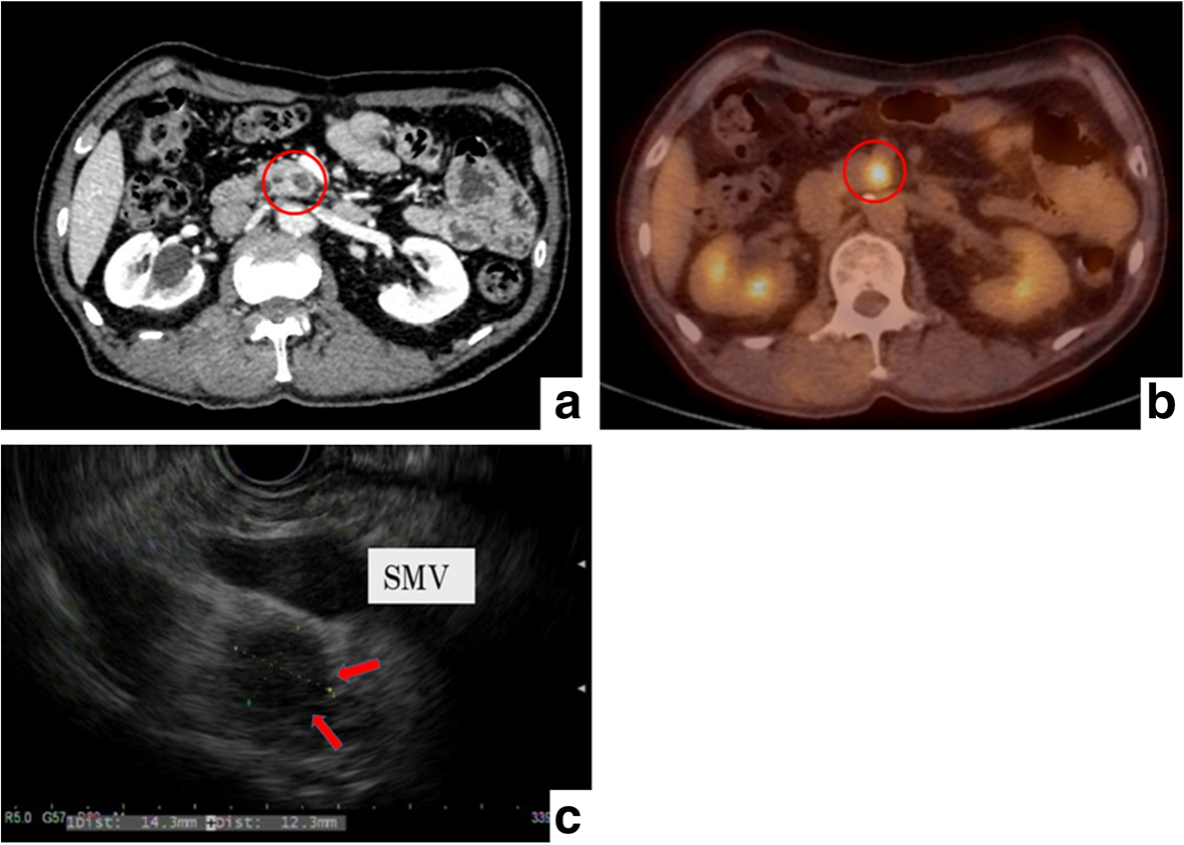
Radiological and endoscopic ultrasonographic findings in case 2. **a** CECT revealed an 8-mm hypovascular tumor in the uncus of the pancreas. **b** PET-CT showed FDG accumulation (SUVmax 5.6) corresponding with the pancreas tumor. **c** EUS detected a 14 × 12-mm hypoechoic lesion in the uncus, but we could not perform EUS-FNA because the superior mesenteric vein was located in the puncture line

**Fig. 4:**
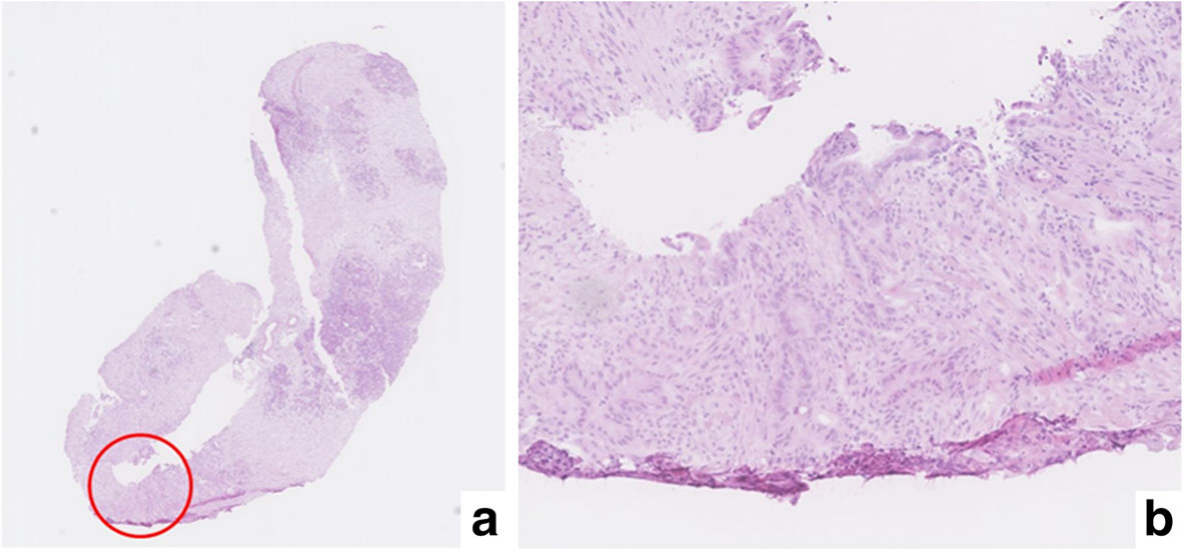
Pathological analysis by open surgical biopsy in case 2. **a** The biopsy specimen had dyskaryotic cells with enlarged nuclei and atypical irregular ductal structures. **b** Microscopic view of specimen (×100 magnification)

Case 2: A 68-year-old man presented to our hospital with elevated CA19-9 levels. The patient had a history of gastric cancer and had been treated by distal gastrostomy and Roux-en Y reconstruction. The patient’s CA19-9 level was 132 U/ml, and CECT revealed a common bile duct stone and an 8-mm hypovascular tumor in the uncus of the pancreas (Fig. 3a). The elevated CA19-9 level was transient, and it suggested that the elevation of CA19-9 had been caused by the inflammation in the bile duct. We then performed a double balloon endoscopic retrograde cholangiopancreatography (DB-ERCP), but we were unable to identify the ampulla of Vater due to the postoperative status of the Roux-en Y reconstruction. PET-CT revealed FDG accumulation (SUVmax 5.6) at the lesion in the uncus (Fig. 3b). EUS detected a 14 × 12 mm hypoechoic lesion in the uncus, but we could not perform EUS-FNA because the superior mesenteric vein was located in the puncture line (Fig. 3c). Therefore, we performed an intraoperative pancreas biopsy. As similar with case 1, we started intraoperative biopsies by 20-gauge needle, but finally we needed 16-gauge needle. The specimen showed abundant dyskaryotic cells with enlarged nuclei and atypical, irregular ductal structures. These observations were consistent with adenocarcinoma (Fig. 4a, b). The patient received preoperative CRT (radiation: 50.5 Gy, chemotherapy: gemcitabine and S-1) and underwent PD. After the operation, the patient’s pathological stage was pT3N1M0 stage IIB. Three months after the operation, follow-up CECT revealed a 2-cm irregular lesion in segment 6 and 7 of the liver, and we diagnosed a tumor recurrence in the liver. Consequently, the patient underwent systemic chemotherapy with gemcitabine and nab-paclitaxel at the frequency once in 2 weeks.

## Conclusions

The ESMO clinical guidelines for cancer of the pancreas state that histological diagnosis by tumor biopsy is indicated for patients before initiating chemotherapy or CRT. EUS-FNA is routinely performed to diagnose pancreatic malignancy. The sensitivity and specificity of EUS-FNA for pancreatic neoplasms are reported as 64–85 and 90–100%, respectively [9, 13], and the diagnostic accuracy is reported as 78–95% [7, 8]. However, FNA usually yields a small volume of tissue, and in some cases, it is insufficient for definitive diagnosis. Moreover, EUS-FNA cannot be performed safely when the artery, portal vein, or main pancreatic duct fall within the puncture route. The success rate of EUS-FNA depends upon the experience of the endoscopists [14]. Recent advances in laparoscopic surgical techniques have enabled ultrasonography-guided laparoscopic biopsy [15], but peritoneal dissemination after biopsy has been observed in some cases. Maemura et al. reported peritoneal dissemination in four out of 25 cases after laparoscopic ultrasonography-guided CNB [16]. And the port site peritoneal metastasis could occur after laparoscopic biopsy for pancreatic cancer. However, ultrasonography-guided laparoscopic biopsy may be useful for unresectable or metastatic pancreatic cancer when methods such as EUS-FNA or percutaneous or CT-guided biopsy have failed to establish a diagnosis. The utility of making a preoperative or pretreatment diagnosis in patients with resectable pancreatic cancer remains controversial. The method of open surgical biopsy is a popular option for the diagnosis of resectable pancreatic cancer after other modalities fail.

Open surgical biopsy for pancreatic neoplasms started to be conducted around 1960. The complication rate, including bleeding, hematoma, wound infection, and pancreatitis, was reported as 6.2–13.6% [17–20], and the sensitivity was 50–78% [19, 20]. In these reports, a 12-gauge Vim–Silverman needle was used to make the puncture. From previous reports, we speculated that thick needle of 12 gauge caused complications after open surgical biopsy. Then, we started the thinnest puncture needle (20 gauge) for first puncture. In another point, we ordered the pathological evaluation for each samples obtained by each procedures, and when tumor samples were insufficient to establish a definitive diagnosis, we re-tried the puncture by thicker needle. In the present report, we did not observe any complications after open surgical biopsy.

There are a number of reports regarding the effectiveness of preoperative CRT for pancreatic cancer [21–23]. Hoffman et al. reported that preoperative CRT followed by surgical resection resulted in tumor-free resection margins and longer survival after the resection. Breslin et al. reported that preoperative CRT combined with PD prolonged survival and lower tumor recurrence rate. Our study group conducted phase I clinical trial of preoperative CRT (gemcitabine and S-1) for resectable pancreatic cancer and demonstrated that the regimen of preoperative CRT was feasible and well tolerated [24]. According to the Evans classification of the therapeutic effects of preoperative CRT, the patients in this report demonstrated pathological antitumor effects of grades IIa and IIb, respectively. Based on the results of the phase I trial, we are now conducting a phase II trial of preoperative CRT for resectable pancreatic cancer. Both of the patients described in this report were enrolled in this phase II trial.

In summary, we report two cases of patients with pancreatic cancer who underwent open surgical biopsy because EUS-FNA failed to yield a definitive pathological diagnosis. EUS-FNA is the first choice of diagnostic modalities for pancreatic neoplasms, but open surgical biopsy can be considered as an effective diagnostic method if EUS-FNA is unsuccessful.

## Abbreviations

18 F-FDG: Fluorine‑18 fluorodeoxyglucose
AJCC: American Joint Committee on Cancer
CA19-9: Carbohydrate antigen 19-9
CEA: Carcinoembryonic antigen
CECT: Contrast-enhanced CT
CNB: Core needle biopsy
CRT: Chemoradiation therapy
CT: Computed tomography
DB-ERCP: Double balloon endoscopic retrograde cholangiopancreatography
DUPAN-2: Duke pancreatic monoclonal antigen type 2
ESMO: European Society for Medical Oncology
EUS-FNA: Endoscopic ultrasound fine-needle aspiration
NCCN guidelines: National Comprehensive Cancer Network Clinical Practice Guidelines in Oncology
PD: Pancreaticoduodenectomy
PET-CT: Positron emission tomography-CT
UICC: Union for International Cancer Control
